# Muscle synergies are consistent across level and uphill treadmill running

**DOI:** 10.1038/s41598-018-24332-z

**Published:** 2018-04-13

**Authors:** Akira Saito, Aya Tomita, Ryosuke Ando, Kohei Watanabe, Hiroshi Akima

**Affiliations:** 1 Graduate School of Arts and Sciences, The University of Tokyo, Komaba, Meguro-ku, Tokyo, Japan; 2 Japan Society for the Promotion of Science, Kojimachi, Chiyoda-ku, Tokyo, Japan; 3 Graduate School of Education and Human Development, Nagoya University, Furo-cho, Chikusa-ku, Nagoya, Aichi Japan; 4 Research Center of Health, Physical Fitness & Sports, Nagoya University, Furo-cho, Chikusa-ku, Nagoya, Aichi Japan; 5 School of International Liberal Studies, Chukyo University, Yagotohonmachi, Showa-ku, Nagoya, Aichi Japan

## Abstract

This study aimed to identify muscle synergies of the lower limb during treadmill running on level and inclined ground. Eight subjects ran on a treadmill at three speeds (2.5, 3.3, and 4.1 m/s) and two grades (level and 10% grade). Surface electromyographic (EMG) signals were recorded from 10 muscles of the lower limb, including deeper muscles such as vastus intermedius, adductor magnus, and adductor longus. Muscle synergies were extracted applying a non-negative matrix factorization algorithm, and relative co-activations across muscles and the temporal recruitment pattern were identified by muscle synergy vector and synergy activation coefficient, respectively. The scalar product between pairs of synergy vectors and synergy activation coefficients during level and uphill running conditions were analyzed as a similarity index, with values above 0.8 recognized as similar. Approximately 4 muscle synergies controlled the majority of variability in 10 EMGs during running, and were common between level and uphill conditions. At each running speed, inter-condition similarity was observed in synergy vector (*r* > 0.83) and synergy activation coefficients (*r* > 0.84) at each type of synergy. These results suggest that types of synergy are consistent between level and uphill running.

## Introduction

Running is one of the modes of human locomotion and is generally considered a distinct locomotor mode with strikingly different mechanics from walking^1^. Locomotor behavior during running usually changes depending on the running speed (i.e., jogging and sprinting)^2^. Moreover, locomotor behaviors change in response to changes in geographical factors, including the surface and slope of the ground^3^. The present study concerns the neuromuscular adaptation of running, to provide a better understanding of the fundamental control of human locomotion.

In locomotion studies, electromyography (EMG) recordings have assessed the temporal patterns of neuromuscular activation in working muscles within a step cycle, and typical EMG activation patterns of the lower limb during running are well documented^4–6^. Neuromuscular activation during running shows non-uniform changes in response to alternations in slope^7–9^. In particular, slope-related changes in EMG activation during high-speed running has been observed in the quadriceps femoris and hip adductors^9^. Regarding EMG recordings for deeper muscles in the thigh, specific activation patterns were obtained from knee and hip muscles, such as vastus intermedius (VI)^6^, adductor magnus (AM)^4^, and adductor longus (AL)^5^ during running over a wide speed range. Neuromuscular activation of the thigh muscles, including deeper muscles, may thus demonstrate the existence of task-specific modulation during running.

In the last decade, neural control strategies for activating muscles during locomotion have been predicted by applying a non-negative matrix factorization (NMF) for the EMG activation patterns of each muscle^10,11^. Locomotor muscle activities are generated by motor neurons in the spinal cord, and spinal central pattern generators are suggested to play an essential role in producing coordinated muscle activities^11,12^. Indirect evidence suggests that the human spinal cord generates rhythmic and synergistic activities in the lower limb^13^. Assuming the existence of the modular control strategies (i.e., muscle synergies), human locomotion might be controlled by a sequence of several motor modules^10^. In fact, a small number of muscle synergies could account for the majority of surface EMG patterns from a large number of muscles while running over a wide speed range using NMF^10,14^. Neuromuscular adaptation of synergies in walking has been addressed between level and inclined surfaces, and these synergies are flexibly used to achieve walking adapted to the mechanical demand^15^. However, little information has been accumulated regarding the modulation of muscle synergies between running conditions with different inclines. Since walking and running are controlled by distinct sets of muscle synergies^14^, this study may show that task-specific modulation of the synergies was induced by inclined running. Specifically, slope-related changes in EMG activity were observed in the initial stance and swing phases of running^9^, corresponding to synergies comprising mainly hip and knee extensors, and hip adductors and knee flexors, respectively^10,14^. We therefore assumed that the synergies activating in the initial stance and swing phases in running are modulated by alternations in slope.

We recorded neuromuscular activations of the lower limb, including the deeper muscles, during running on level and inclined ground, then extracted muscle synergies using NMF. Extraction of muscle synergies was performed at three different running speeds on the treadmill to evaluate the dependence of synergy on speed. This study aimed to test the hypothesis that muscle synergies mainly comprising activation of knee extensors and/or hip adductors during running are modulated in response to ground elevation.

## Method

### Subjects

Seven men and one woman were recruited for the present study. The respective physical characteristics of subjects were as follows: age, 26.5 ± 6.9 years; height, 167.3 ± 9.6 cm; and body mass, 59.1 ± 12.8 kg. The procedure, purpose, risks, and benefits associated with the present study were explained to subjects and written informed consent was obtained prior to the experiment. The institutional review board of the Research Center of Health, Physical Fitness & Sports at Nagoya University approved the experimental protocols, which were conducted in accordance with the guidelines of the Declaration of Helsinki.

### Experimental protocol

Subjects ran on a motorized treadmill (TREAD-MILL; Nishikawa Iron Works, Kyoto, Japan) set to 1.6 m/s and level (i.e., 0° incline) for 3 min prior to testing. Subjects completed a 1-min running trial on the treadmill set to 6 different combinations of speed and grade; i.e., 2.5, 3.3, and 4.1 m/s either on the level or on a grade of 10%. Speed-grade combinations were conducted in randomized order for each subject. The rest interval between each trial was at least 1 min.

### Surface EMG recording

Surface EMG signals were recorded during running from ten muscles of the right lower limb: vastus intermedius (VI), vastus lateralis (VL), vastus medialis (VM), rectus femoris (RF), the long-head of biceps femoris (BF), semimembranosus (SM), adductor magnus (AM), adductor longus (AL), the medial head of gastrocnemius (MG), and tibialis anterior (TA). EMG sensors consisting of two silver bar electrodes (0.1 × 1 cm each), with a 1-cm inter-electrode distance, were used for EMG acquisition from each muscle. A DE-2.1 sensor pre-amplifier and main amplifier were set with a bandpass filter at 20–450 Hz (Bagnoli; Delsys, Boston, USA). Signals were sampled at 2000 Hz using an AD converter (Power Lab; ADInstruments, Melbourne, Australia). Timings of heel contact were identified by a foot-switch (DL-250; S&ME, Tokyo, Japan) taped to the heel of a right shoe. We determined the gait cycle using the foot-switch attached to the heel for EMG analysis. One heel contact to next heel contact was defined as one gait step. Electrical signals from the foot-switch were synchronized with surface EMG signals on a personal computer using Chart 7 software (ADInstruments).

After shaving, abrading, and cleaning the skin with alcohol, electrodes were positioned at specific locations for each muscle^16^. We determined the superficial regions of the VI, AL, and AM using ultrasonography (Logiq e; GE Healthcare, Duluth, USA)^17,18^. The VI electrode was positioned on the skin where the superficial region of the VI overlapped at 0° and 65° of knee flexion^19–23^. Electrodes for AM and AL were placed on the skin between the SM and gracilis and sartorius, respectively^18^. A reference electrode was placed over the iliac crest.

### Post-processing and extraction of muscle synergies

During the stable running of each trial, 10 consecutive gait cycles were sampled for analysis. Based on the variability of EMG magnitude and heel-to-heel contact cadence while running, the stable running phase was visually determined. EMG signals were rectified and smoothed with a low-pass filter at 15 Hz using a fourth-order Butterworth filter^24,25^. EMG envelopes of each cycle were interpolated to 100 time points. EMG patterns were normalized to the respective maximum amplitude across all speeds and inclinations.

We extracted muscle synergies for each subject using NMF^26^. The EMG data matrix for each condition and speed was averaged across consecutive 10 cycles^10,24^. NMF minimizes the residual between initial matrix and its decomposition, given as follows (1):

1 $$E=WH+e,$$

where E is a *p* × *n* matrix (where *p* is number of muscles and *n* is the number of time points), *W* is a *p* × *k* matrix of the synergy vectors, containing the spatial information of muscle coactivations, H is a *k* × *n* matrix of the synergy activation coefficients involving the temporal information of synergy recruitment, *k* is the number of extracted muscle synergies, and *e* is the residual error matrix. Muscle synergy vectors were normalized by the maximum under the synergy to which they belong. The peak phase of synergy activation coefficients was identified from among 100 time points and expressed as percentage of cycle.

We iterated analyses by varying the number of synergies between 1 and 10, then the least number of synergies *k* that accounted for >90% of the variance accounted for (VAF) in each subject was selected^27^ and VAF was defined as the uncentered Pearson correlation coefficient (2):

2 $${\rm{VAF}}=1-\frac{\sum _{i=1}^{p}\sum _{j=1}^{n}{({e}_{i,j})}^{2}}{\sum _{i=1}^{p}\sum _{j=1}^{n}{({E}_{i,j})}^{2}}$$

### Inter-subject similarities of muscle synergies

To perform functional sorting of extracted muscle synergies, we initially performed classification by grouping muscle synergies based on the cosine similarity value (*r* > 0.60) with that of an arbitrary reference subject using an iterative process^28^. The averaged set of muscle weight components for all subjects was then computed, and the cosine similarity between averaged muscle synergies and each synergy across subjects was calculated^29^. If two synergies in one subject were assigned to the same synergy, the pair of synergies with the highest correlation was defined as the same group of synergies.

### Similarities of muscle synergies

We quantified the similarity of synergy vectors and activation coefficients between running speeds and between level and uphill running conditions by computing scalar products adjusted for the delay^30,31^. More specifically, the delay in a pair of synergy activation coefficients was assessed as lag time at the maximum of the cross-correlation function^32,33^. Inter-condition similarity was then calculated by computing the scalar product between pairs of vectors (i.e., synergy vectors or synergy activation coefficients), normalized by the product of the norms of each column^30^, to prioritize the comparison between shapes of vectors rather than amplitude^31^. Similarity can vary from 0 (no curve shape matching) to 1 (perfect curve shape matching), and values above 0.8 were defined as indicating similarity between a pair of vectors^34^.

### Statistics

Since numbers of samples within the muscle synergy differed between synergy types or running conditions and these data showed partly non-Gaussian distributions, we performed non-parametric testing to compare the difference between running conditions and speeds. Cadence, peak amplitude at each EMG, number of synergies, and weighting components of synergy between level and uphill running were analyzed using the Wilcoxon signed-rank test. These variables between different running speeds were analyzed using the Friedman test. If a significant difference was identified, the Wilcoxon signed-rank test was performed as a post hoc test. Furthermore, differences in peak timing of EMG activity and synergy activation coefficients between running conditions and speed were analyzed using the Watson-Williams test using a toolbox^35^ for MATLAB (version R2016a; Mathworks, Natick, USA). The level of statistical significance was set at the *p* < 0.05, and *p* value for multiple comparisons was adjusted by Bonfferoni correction.

### Data availability

The datasets generated during and/or analysed during the current study are available from corresponding author on reasonable request.

## Results

Running cadence (i.e., right-leg heel contacts) at 2.5, 3.3, and 4.1 m/s were 85.0 ± 5.0, 88.8 ± 5.5, and 94.1 ± 6.6 steps/min, respectively, during level running and 86.6 ± 5.3, 91.3 ± 6.4, and 96.8 ± 9.7 steps/min, respectively, during uphill running. No significant differences in cadence were observed between running conditions at each speed (*p* > 0.05). Cadence increased significantly with increasing running speed under each running condition (*p* < 0.05).

**Figure 1:**
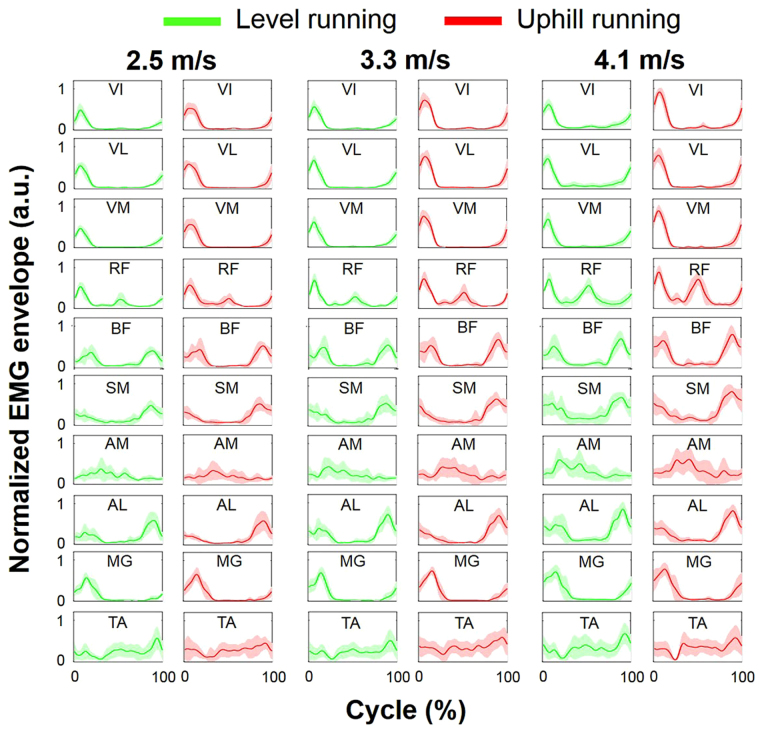
Ensemble-averaged electromyographic (EMG) patterns of 10 recorded muscles for each condition. Averaged EMG patterns across 10 consecutive running cycles were normalized to the respective maximum value among all speeds and inclinations and expressed as a function of the percentage of the cycle. Colored bold lines indicate mean EMG patterns and colored filled areas indicate the standard deviation. a.u., arbitrary units.

Peak EMG activity in the lower limb increased significantly with increasing running speed for each condition (*p* < 0.05), with the exception of MG during uphill running and TA under each running condition (*p* > 0.05) (Fig. 1). Peak EMG activity of the VM was significantly higher during uphill running than during level running at each speed, and that of the VI was significantly higher during uphill running than during level running at 4.1 m/s (*p* < 0.05). No significant difference in the timing of peak EMG activation was observed between running conditions and between speeds for each muscle (*p* > 0.05).

### Properties of muscle synergies

**Figure 2:**
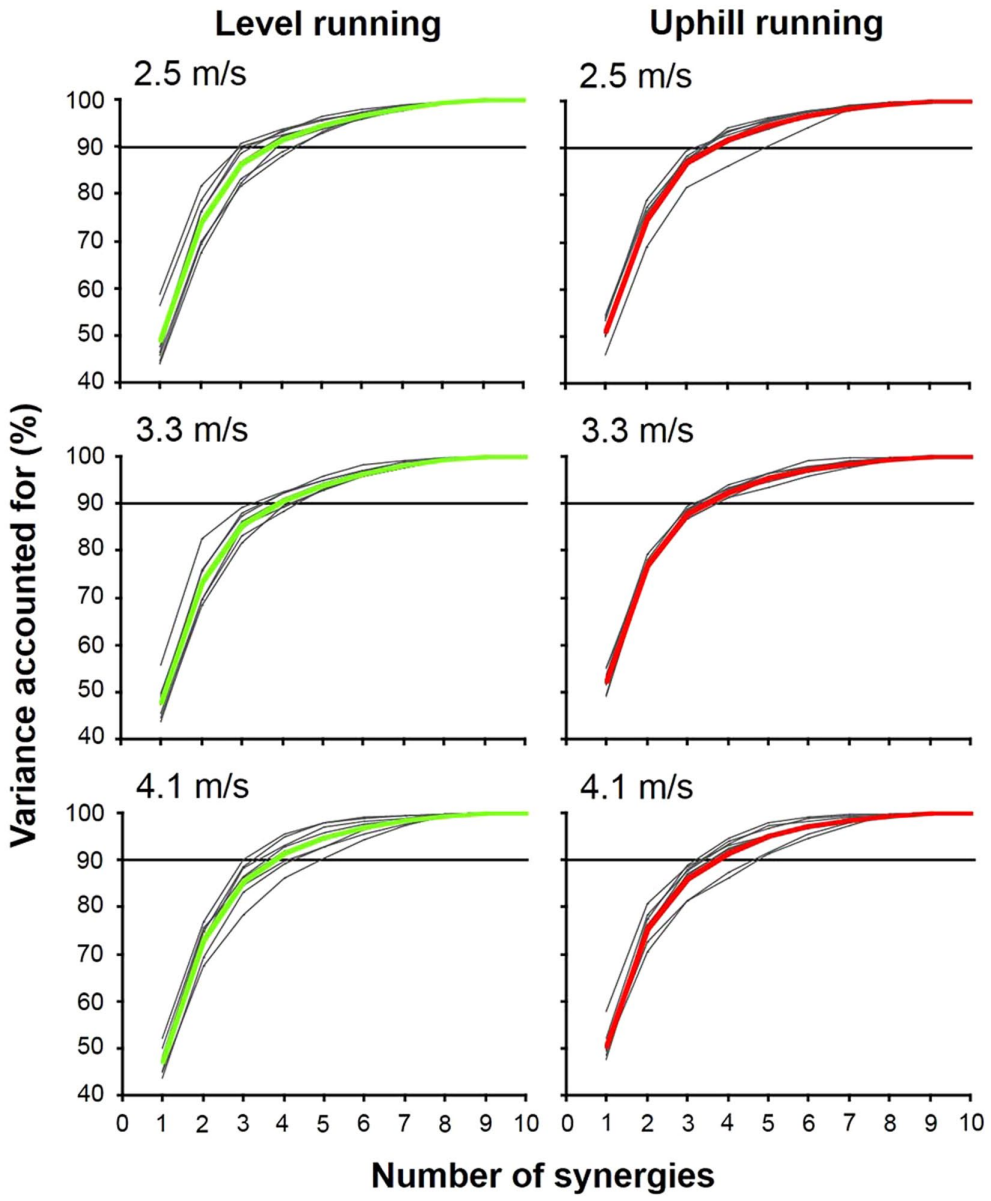
Variance accounted for (VAF) as a determination of the number of extracted muscle synergies under each running condition. Iterative analysis was performed by varying the number of synergies between one and ten, and the least number of synergies that accounted for >90% VAF in each subject was identified (gray narrow lines). Colored bold lines indicate mean VAFs across subjects, with 90% VAF shown as a solid black line.

Based on cumulative percentages of variance explained by each muscle synergy for each condition, three to five types of muscle synergy were identified across all subjects (Fig. 2). Mean numbers of synergies during level running at 2.5, 3.3, and 4.1 m/s were 4.1 ± 0.6, 4.3 ± 0.5, and 4.2 ± 0.7, respectively, and those during uphill running at 2.5, 3.3, and 4.1 m/s were 4.1 ± 0.3, 4.0 ± 0.0, and 4.2 ± 0.4, respectively (VAF > 90%). No significant difference in number of synergies was evident between running conditions and between speeds (*p* > 0.05).

**Figure 3:**
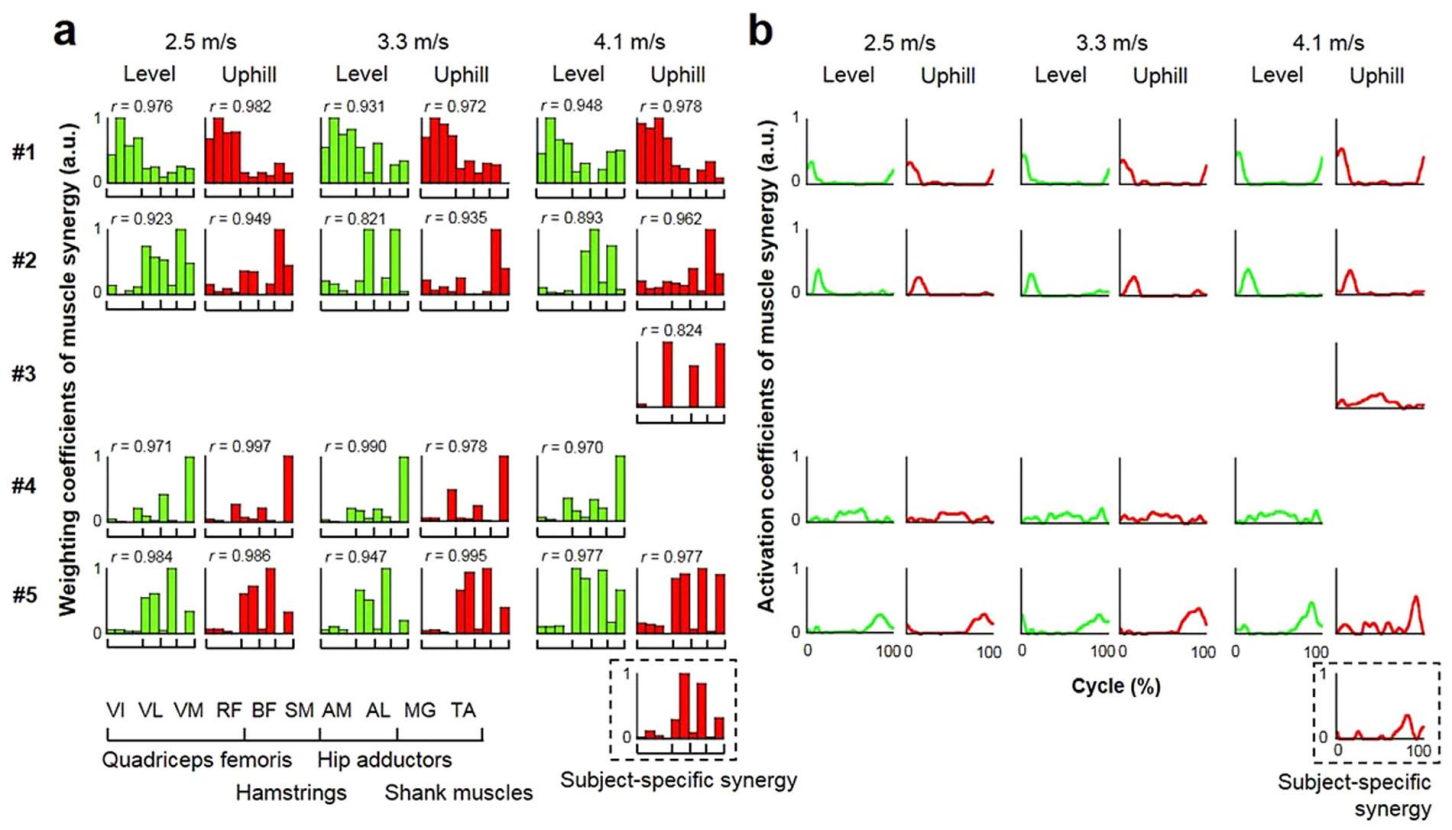
A typical example of extracted muscle synergy during level and uphill running. (**a**) Each bar represents the relative spatial information of muscle coactivation within each synergy. Weighting coefficients were normalized by maximum values under the synergy. The *r* value represents cosine similarities between averaged muscle weighting components across subjects, and is shown just above each bar graph. (**b**) Each waveform represents the temporal activation pattern of the synergy related to individual muscle weighting components. Synergy surrounded by a dashed line is a subject-specific synergy. #1, Synergy 1; #2, Synergy 2; #3, Synergy 3; #4, Synergy 4; #5, Synergy 5; a.u., arbitrary units.

Muscle synergies were classified into similar groups and sorted across subjects. Figure 3 demonstrates a typical example of synergies consisting of synergy weighting components (Fig. 3a) and the associated temporal activation patterns (Fig. 3b) under six different combinations of three speeds and two grades. In that subject, four or five types of muscle synergies were extracted under each running condition. Weighting components in five types of synergies were similar across subjects (0.821 < *r* < 0.997), whereas one synergy in uphill running at 4.1 m/s was inconsistent with the synergy in other subjects (i.e., subject-specific synergy) (Fig. 3).

**Figure 4:**
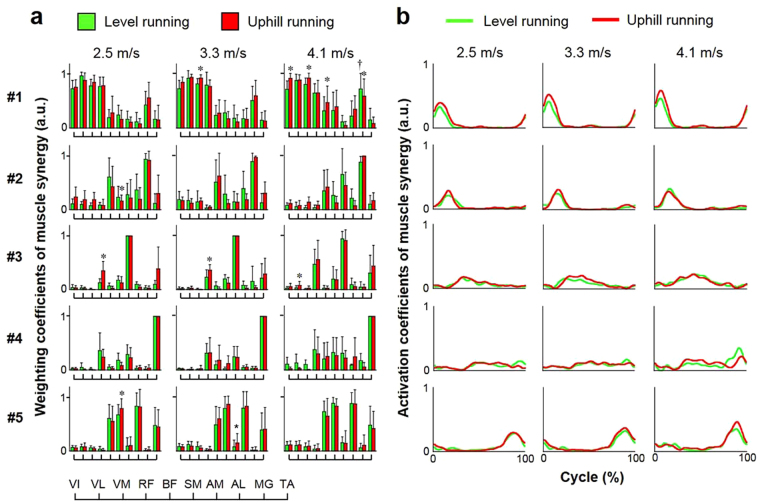
Averaged muscle synergies during level and uphill running across all subjects. (**a**) Bar graphs indicate five types of weighting components of muscle synergies normalized by maximum values under that synergy. Colored bars indicate the mean of normalized weighting components and black bars indicate the standard deviation. ^*^p < 0.05, vs. level running; ^†^*p* < 0.05, vs. 2.5 m/s. (**b**) Line graphs express five types of activation coefficients of muscle synergies. #1, Synergy 1; #2, Synergy 2; #3, Synergy 3; #4, Synergy 4; #5, Synergy 5; a.u., arbitrary units.

We found five types of muscle synergy that were common among all subjects during level and uphill running (Table 1, Fig. 4). The number of samples in muscle synergies which shared among subjects was influenced by synergy type and running condition, and some subject-specific synergies were observed under each condition (Table 1). *Synergy 1* consisted mainly of activation of the quadriceps femoris (0.898 < *r* < 0.992), which was activated with the timing of right-heel contact. *Synergy 2* mainly recruited MG (0.814 < *r* < 0.979) and was activated following the activation of *Synergy 1*. *Synergy 3* and *Synergy 4* mainly consisted of AM (0.796 < *r* < 0.996) and TA (0.836 < *r* < 0.997), respectively, and were activated during the later stance or swing phases. *Synergy 5* consisted primarily of activation of the AL and two muscles of the hamstrings (0.749 < *r* < 0.995), and was activated before the timing of right-heel contact.

**Table 1:** Properties of muscle synergy and number of subjects in each type of synergy.

Synergy type	Major muscles	Level running			Uphill running		
		2.5 m/s	3.3 m/s	4.1 m/s	2.5 m/s	3.3 m/s	4.1 m/s
Synergy 1	VI, VL, VM, RF	8	8	8	7	8	8
Synergy 2	MG	7	6	5	5	4	5
Synergy 3	AM	5	6	6	5	5	7
Synergy 4	TA	4	4	4	5	6	3
Synergy 5	BF, SM, AL	8	8	8	8	8	8
Subject-specific	—	1	3	3	3	1	3

### Muscle synergies between running speeds

Weighting components in five types of muscle synergies were similar between different running speeds at each condition (*r* > 0.84), except for that in *Synergy 2* at level running (3.3 vs. 4.1 m/s) (Table 2). Activation coefficients in the five types of synergy were similar between speeds under each condition (*r* > 0.80). With comparison of the weighting components between different running speeds, a significant change in weighting components was observed only in the MG within *Synergy 1* (*p* < 0.05) (Fig. 4). No significant difference in peak timing of each synergy activation coefficient was observed between running speeds (*p* > 0.05).

**Table 2:** Similarity of muscle synergies across different speeds.

	Level running			Uphill running		
	2.5 vs. 3.3 m/s	3.3 vs. 4.1 m/s	2.5 vs. 4.1 m/s	2.5 vs. 3.3 m/s	3.3 vs. 4.1 m/s	2.5 vs. 4.1 m/s
**Synergy vectors**						
Synergy 1	0.96 ± 0.02	0.92 ± 0.04	0.93 ± 0.04	0.98 ± 0.01	0.95 ± 0.05	0.94 ± 0.04
Synergy 2	0.88 ± 0.09	0.76 ± 0.04	0.84 ± 0.09	0.92 ± 0.04	0.92 ± 0.05	0.87 ± 0.07
Synergy 3	0.95 ± 0.06	0.95 ± 0.05	0.88 ± 0.09	0.98 ± 0.01	0.97 ± 0.01	0.94 ± 0.04
Synergy 4	0.96 ± 0.01	0.95 ± 0.03	0.96 ± 0.01	0.97 ± 0.01	0.87 ± 0.07	0.88 ± 0.11
Synergy 5	0.93 ± 0.08	0.94 ± 0.05	0.91 ± 0.07	0.97 ± 0.01	0.95 ± 0.04	0.92 ± 0.08
**Synergy activation coefficients**						
Synergy 1	0.97 ± 0.02	0.95 ± 0.02	0.95 ± 0.02	0.97 ± 0.02	0.96 ± 0.02	0.95 ± 0.05
Synergy 2	0.94 ± 0.02	0.91 ± 0.03	0.92 ± 0.02	0.95 ± 0.02	0.95 ± 0.02	0.92 ± 0.04
Synergy 3	0.92 ± 0.04	0.93 ± 0.04	0.89 ± 0.05	0.93 ± 0.02	0.94 ± 0.03	0.92 ± 0.03
Synergy 4	0.89 ± 0.05	0.87 ± 0.11	0.80 ± 0.09	0.90 ± 0.07	0.83 ± 0.04	0.89 ± 0.03
Synergy 5	0.93 ± 0.04	0.90 ± 0.07	0.87 ± 0.10	0.95 ± 0.05	0.91 ± 0.10	0.93 ± 0.05

Values are mean and standard deviation. Similarity of muscle synergies for each subject was computed by the scalar products after adjusting for the delay.

### Muscle synergies between running conditions

**Figure 5:**
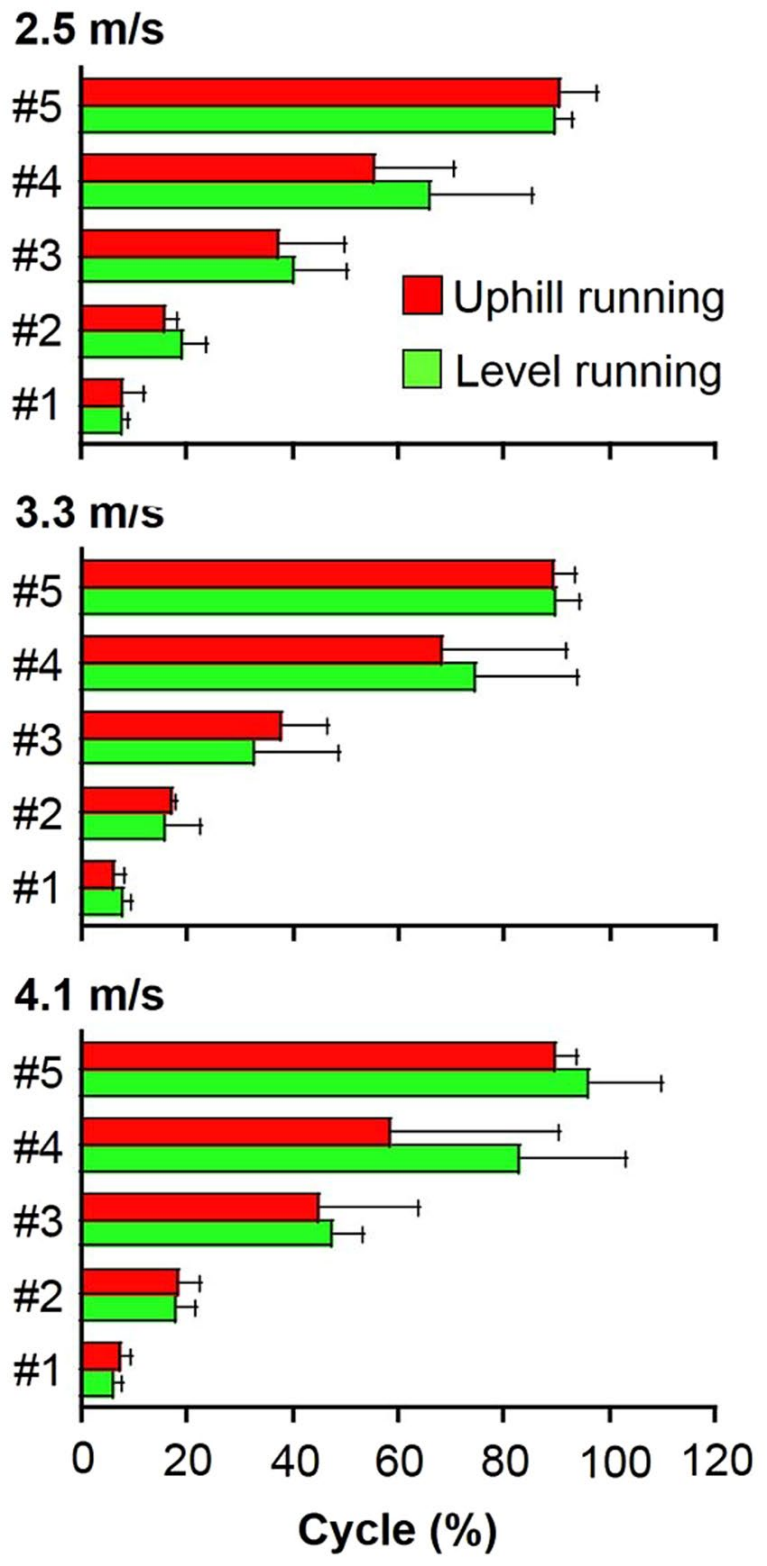
Peak activation timing (% of cycle) for the five types of muscle synergy during level and uphill running. Colored bars indicate mean peak timing and black bars indicate the standard deviation. ^*^p < 0.05, vs. level running. #1, Synergy 1; #2, Synergy 2; #3, Synergy 3; #4, Synergy 4; #5, Synergy 5

Weighting components in five types of muscle synergies were similar between level and uphill running conditions at each speed (*r* > 0.83) (Table 3). Activation coefficients in the five types of synergy were also similar between conditions at each speed (*r* > 0.84). Although characteristics of muscle synergies were common to both level and uphill running, several synergy weighting components and peak timing of the synergy activation coefficient were significantly different (Figs 4 and 5). In particular, a significant difference in the weightings of VI, VM, BF, and MG in *Synergy 1* was observed between level and uphill conditions at 4.1 m/s (*p* < 0.05) (Fig. 4a). No significant difference between level and uphill conditions was observed in peak timing of each synergy activation coefficient (Fig. 5).

**Table 3:** Inter-condition similarity of muscle synergies.

	2.5 m/s	3.3 m/s	4.1 m/s
**Synergy vectors**			
Synergy 1	0.94 ± 0.03	0.93 ± 0.04	0.95 ± 0.02
Synergy 2	0.88 ± 0.09	0.83 ± 0.12	0.87 ± 0.09
Synergy 3	0.89 ± 0.10	0.96 ± 0.03	0.84 ± 0.24
Synergy 4	0.97 ± 0.01	0.97 ± 0.01	0.86 ± 0.12
Synergy 5	0.94 ± 0.05	0.96 ± 0.02	0.95 ± 0.06
**Synergy activation coefficients**			
Synergy 1	0.97 ± 0.02	0.96 ± 0.03	0.97 ± 0.01
Synergy 2	0.90 ± 0.08	0.95 ± 0.01	0.94 ± 0.03
Synergy 3	0.92 ± 0.03	0.96 ± 0.01	0.84 ± 0.24
Synergy 4	0.84 ± 0.18	0.94 ± 0.02	0.90 ± 0.05
Synergy 5	0.91 ± 0.09	0.89 ± 0.15	0.88 ± 0.12

Values are mean and standard deviation. Inter-condition similarity of muscle synergies for each subject was computed by the scalar products after adjusting for the delay.

## Discussion

Neuromuscular activations of the lower limb including the deeper muscles were recorded during running on level and inclined ground surfaces, then muscle synergies were identified by NMF. This study showed that three to five muscle synergies accounted for the majority of activation profiles in 10 lower limb muscles for each subject. Moreover, inter-condition similarities were observed in weighting components and activation coefficients for each type of synergy. These results did not support the hypothesis that synergies mainly comprising activation of knee extensors and/or hip adductors during running were modulated in response to ground elevation.

Previous studies have demonstrated that several muscle synergies control the total patterns of a large number of neuromuscular activation in the lower limb and trunk muscles during running^10,14,36,37^. In this study, three to five types of muscle synergy provided > 90% of the total VAF for each subject (Fig. 2). Accordingly, approximately 4 muscle synergies controlled the majority of the 10 EMG profiles under each running condition. Cappellini *et al*.^10^ demonstrated that five types of components primarily control human running, and these components were activated at the specific phases of running, i.e., initial-stance (*Component 1*, hip and knee extensors), middle-stance (*Component 2*, plantar flexors), initial-swing (*Component 3*, erector spinae and upper trunk), middle-swing (*Component 4*, dorsiflexors), and late-swing to early stance (*Component 5*, hip adductors and hamstrings). These five components globally corresponded to the specific type of synergy in this study (Table 1, Fig. 4). Under the muscle synergy hypothesis, the motor program in human running is speculated to be a sequence of several temporal activation of synergies^10,14^. Our results thus suggest that three to five temporal activation patterns of muscle synergies principally control locomotor muscle activities in the lower limb during running.

By comparing muscle synergies between level and uphill running conditions at each speed, the results of this study showed global consistency in the synergies across conditions. First, mean numbers of muscle synergies for all subjects were similar across the conditions at each running speed. Second, we confirmed inter-condition similarities of synergy vectors (*r* > 0.83) and synergy activation coefficients (*r* > 0.84) in each type of synergy (Table 3). These results suggest that basic patterns of locomotor muscle activity are consistent between level and uphill running. Regarding the motor modules controlling human running under different environmental conditions, Oliveira *et al*.^36^ suggested that similar motor modules control human running under varying environmental requirements (treadmill vs. overground running). Ivanenko *et al*.^38^ demonstrated that specific components were additionally recruited to five basic components when subjects added a voluntary task (e.g., kick a ball and step over an obstacle) to their normal walking. We therefore consider that the recruited synergies in the lower limb were shared across different running conditions, because the natural motor behavior of running was performed for individual subjects regardless of the conditions.

Although the weighting components and temporal activation patterns of muscle synergies were similar between level and uphill running, differences between conditions were observed in muscles within a synergy (Fig. 4). Differences in knee extensor weightings in *Synergy 1* across conditions corresponded to the peak EMG responses of VI and VM during uphill running, supporting previous findings^9^. These differences in other synergies may be induced by changes in running motions with ground elevation (e.g., contact time and step length^3^). Furthermore, Janshen *et al*.^15^ reported modulation of muscle synergies between level and uphill walking during the stance phase and in the preparation for the stance phase, corresponding to the change with vertical force in the uphill condition. In practical terms, foot strike modes of running modulate the weightings of muscle synergies^37,39^. More specifically, changes in foot strike pattern induced modulation of the weightings of knee extensors and plantar flexors within the synergy recruited during the propulsion phase of running^37^. We thus considered that the neuromuscular activation and manner of running caused by changing the grade and mechanical demand would be factors in the modulation of weighting components observed in any muscles within the synergy.

Temporal activation patterns of each type of synergy were similar between running conditions (Fig. 5). A previous study showed that time shifts in synergy activation coefficients were less than 4% of the step cycle between level and 10% grade uphill walking conditions^32^. Saito *et al*.^40^ also demonstrated that the shapes of synergy activation coefficients appeared similar between level and 10% uphill walking, with a few time-shift adjustments with changes in grade (i.e., <8.8% of step cycle). We therefore speculated that the temporal activation pattern of muscle synergy during running was preserved regarding changes in the slope of the ground surface.

Similarities of synergy vectors and synergy activation coefficients in each type of synergy between different running speeds were observed at each running condition (Table 2). Thus, the results in this study suggest that basic patterns of locomotor muscle activity are consistent across running speeds for each condition. Several previous studies compared muscle synergies among running speeds^10,14,39^. Cappellini *et al*.^10^ demonstrated that human running was accomplished by similar basic activation patterns with an increase in speed. We therefore consider muscle synergies as independent of running speed within the ranges of this study (i.e., jogging to running).

Methodological limitations to muscle synergy identification during running must be considered in this study. We recorded locomotor muscle activity from 10 EMGs in the lower limb, and the number of recorded muscles in this study was within the previously reported range (i.e., 8–31 EMGs)^36,41,42^. However, a previous study demonstrated that extracted muscle synergies using an NMF algorithm were sensitive to the number and function of muscles^41^. Hence, possible differences in synergies may be observed if EMGs are collected from large numbers of muscles. Second, previous study recommended that muscle synergy extractions are performed from 20 consecutive gait cycles to include the step-by-step variability^31^, though we used the averaged EMG patterns across gait 10 cycles to identify the synergies based on Ivanenko and colleagues’ work^10,24^. Third, the time scale of each EMG data set was interpolated to 100 time points, less than in previous works^10,14,15^. Since choice of cut-off frequency for the filter for the linear envelope influences the number of muscle synergies^43,44^, the time-interpolation in this study might induce excessive filtering out of EMG data. Finally, in this study, extracted muscle synergies were classified into similar groups and sorted across subjects, based on the previously described method^28,29^. Consequently, the number of subjects within each type of muscle synergy differed across synergy types and running conditions, and common types of synergy across subjects were observed in at least three to five subjects under each running condition (Table 1). This was considered one of the methodological limitations in the comparison of each type of synergy between level and uphill conditions.

In conclusion, we demonstrated that appropriately four muscle synergies could account for the majority of 10 EMG activities of the lower limb during level and uphill treadmill running. Inter-condition similarities were observed in each type of synergy at each running speed, with only minor modulation across different running tasks.

## References

[1] Full RJ, Koditschek DE (1999). Templates and anchors: neuromechanical hypotheses of legged locomotion on land. J. Exp. Biol..

[2] Novacheck TF (1998). The biomechanics of running. Gait Posture.

[3] Vernillo, G. *et al*. Biomechanics and physiology of uphill and downhill running. *Sports. Med*. 10.1007/s40279-016-0605-y (2016). 10.1007/s40279-016-0605-y 27501719

[4] Gazendam MG, Hof AL (2007). Averaged EMG profiles in jogging and running at different speeds. Gait Posture.

[5] Mann RA, Moran GT, Dougherty SE (1986). Comparative electromyography of the lower extremity in jogging, running, and sprinting. Am. J. Sports Med..

[6] Montgomery WH, Pink M, Perry J (1994). Electromyographic analysis of hip and knee musculature during running. Am. J. Sports Med..

[7] Wall-Scheffler CM, Chumanov E, Steudel-Numbers K, Heiderscheit B (2010). Electromyography activity across gait and incline: The impact of muscular activity on human morphology. Am. J. Phys. Anthropol..

[8] Swanson SC, Caldwell GE (2000). An integrated biomechanical analysis of high speed incline and level treadmill running. Med. Sci. Sports Exerc..

[9] Yokozawa T, Fujii N, Ae M (2007). Muscle activities of the lower limb during level and uphill running. J. Biomech..

[10] Cappellini G, Ivanenko YP, Poppele RE, Lacquaniti F (2006). Motor patterns in human walking and running. J. Neurophysiol..

[11] Lacquaniti F, Ivanenko YP, Zago M (2012). Patterned control of human locomotion. J. Physiol. (London).

[12] Kiehn O (2016). Decoding the organization of spinal circuits that control locomotion. Nat. Rev. Neurosci..

[13] Gerasimenko Y (2010). Novel and direct access to the human locomotor spinal circuitry. J. Neurosci..

[14] Yokoyama H, Ogawa T, Kawashima N, Shinya M, Nakazawa K (2016). Distinct sets of locomotor modules control the speed and modes of human locomotion. Sci. Rep..

[15] Janshen L, Santuz A, Ekizos A, Arampatzis A (2017). Modular control during incline and level walking in humans. J. Exp. Biol..

[16] Winter DA, Yack HJ (1987). EMG profiles during normal human walking: stride-to-stride and inter-subject variability. Electroencephalogr. Clin. Neurophysiol..

[17] Watanabe K, Akima H (2009). Cross-talk from adjacent muscle has a negligible effect on surface electromyographic activity of vastus intermedius muscle during isometric contraction. J. Electromyogr. Kinesiol..

[18] Watanabe K, Katayama K, Ishida K, Akima H (2009). Electromyographic analysis of hip adductor muscles during incremental fatiguing pedaling exercise. Eur. J. Appl. Physiol..

[19] Saito A, Watanabe K, Akima H (2015). Coordination among thigh muscles including the vastus intermedius and adductor magnus at different cycling intensities. Hum. Mov. Sci..

[20] Akima H, Saito A (2013). Inverse activation between the deeper vastus intermedius and superficial muscles in the quadriceps during dynamic knee extensions. Muscle Nerve.

[21] Saito A, Akima H (2013). Knee joint angle affects EMG-force relationship in the vastus intermedius muscle. J. Electromyogr. Kinesiol..

[22] Saito A, Watanabe K, Akima H (2013). The highest antagonistic coactivation of the vastus intermedius muscle among quadriceps femoris muscles during isometric knee flexion. J. Electromyogr. Kinesiol..

[23] Akima H, Saito A (2013). Activation of quadriceps femoris including vastus intermedius during fatiguing dynamic knee extensions. Eur. J. Appl. Physiol..

[24] Ivanenko YP, Poppele RE, Lacquaniti F (2004). Five basic muscle activation patterns account for muscle activity during human locomotion. J. Physiol. (London).

[25] Hagio S, Fukuda M, Kouzaki M (2015). Identification of muscle synergies associated with gait transition in humans. Front. Hum. Neurosci..

[26] Lee DD, Seung HS (1999). Learning the parts of objects by non-negative matrix factorization. Nature.

[27] Torres-Oviedo G, Macpherson JM, Ting LH (2006). Muscle synergy organization is robust across a variety of postural perturbations. J. Neurophysiol..

[28] Hagio S, Kouzaki M (2014). The flexible recruitment of muscle synergies depends on the required force-generating capability. J. Neurophysiol..

[29] Torres-Oviedo G, Ting LH (2007). Muscle synergies characterizing human postural responses. J. Neurophysiol..

[30] d’Avella A, Saltiel P, Bizzi E (2003). Combinations of muscle synergies in the construction of a natural motor behavior. Nat. Neurosci..

[31] Oliveira AS, Gizzi L, Farina D, Kersting UG (2014). Motor modules of human locomotion: influence of EMG averaging, concatenation, and number of step cycles. Front. Hum. Neurosci..

[32] Gonzalez-Vargas J (2015). A predictive model of muscle excitations based on muscle modularity for a large repertoire of human locomotion conditions. Front. Comput. Neurosci..

[33] Hug F, Turpin NA, Couturier A, Dorel S (2011). Consistency of muscle synergies during pedaling across different mechanical constraints. J. Neurophysiol..

[34] Oliveira AS, Silva PB, Lund ME, Kersting UG, Farina D (2013). Fast changes in direction during human locomotion are executed by impulsive activation of motor modules. Neuroscience.

[35] Berens P (2009). CircStat: A MATLAB toolbox for circular statistics. J. Stat. Softw..

[36] Oliveira AS, Gizzi L, Ketabi S, Farina D, Kersting UG (2016). Modular control of treadmill vs overground running. PLoS One.

[37] Santuz A, Ekizos A, Janshen L, Baltzopoulos V, Arampatzis A (2017). The influence of footwear on the modular organization of running. Front. Physiol..

[38] Ivanenko YP, Cappellini G, Dominici N, Poppele RE, Lacquaniti F (2005). Coordination of locomotion with voluntary movements in humans. J. Neurosci..

[39] Nishida K, Hagio S, Kibushi B, Moritani T, Kouzaki M (2017). Comparison of muscle synergies for running between different foot strike patterns. PLoS One.

[40] Saito A, Tomita A, Ando R, Watanabe K, Akima H (2017). Similarity of muscle synergies extracted from the lower limb including the deep muscles between level and uphill treadmill walking. Gait Posture.

[41] Steele KM, Tresch MC, Perreault EJ (2013). The number and choice of muscles impact the results of muscle synergy analyses. Front. Comput. Neurosci..

[42] Clark DJ, Ting LH, Zajac FE, Neptune RR, Kautz SA (2010). Merging of healthy motor modules predicts reduced locomotor performance and muscle coordination complexity post-stroke. J. Neurophysiol..

[43] Santuz A, Ekizos A, Janshen L, Baltzopoulos V, Arampatzis A (2017). On the methodological implications of extracting muscle synergies from human locomotion. Int. J. Neural. Syst..

[44] Hug F (2011). Can muscle coordination be precisely studied by surface electromyography?. J. Electromyogr. Kinesiol..

